# Structure–Function Insights into Frog Skin Peptides Reveal Potent Inhibition of West Nile Virus Entry

**DOI:** 10.3390/ijms262010148

**Published:** 2025-10-18

**Authors:** Carla Zannella, Annalisa Chianese, Rosa Giugliano, Valeria Stefanizzi, Alessandra Monti, Nunzianna Doti, Emilia Palazzotto, Floriana Bonura, Giovanni M. Giammanco, Antonio Mastino, Simona De Grazia, Francesca Marino-Merlo, Massimiliano Galdiero, Anna De Filippis

**Affiliations:** 1 Department of Experimental Medicine, University of Campania Luigi Vanvitelli, 80138 Naples, Italy; carla.zannella@unicampania.it (C.Z.); annalisa.chianese@unicampania.it (A.C.); rosa.giugliano@unicampania.it (R.G.); 2 Department of Veterinary Medicine and Animal Production, University of Naples Federico II, 80137 Naples, Italy; 3 Department of Chemical, Biological, Pharmaceutical, and Environmental Sciences, University of Messina, 98166 Messina, Italy; valeria.stefanizzi@unime.it (V.S.); fmarino@unime.it (F.M.-M.); 4 Institute of Biostructures and Bioimaging (IBB), National Research Council (CNR), 80131 Naples, Italy; alessandra.monti@ibb.cnr.it (A.M.); nunzianna.doti@cnr.it (N.D.); 5 Department of Health Promotion, Mother and Child Care, Internal Medicine and Medical Specialties “G. D’Alessandro”, University of Palermo, 90127 Palermo, Italy; emilia.palazzotto@unipa.it (E.P.); floriana.bonura@unipa.it (F.B.); giovanni.giammanco@unipa.it (G.M.G.); simona.degrazia@unipa.it (S.D.G.); 6 The Institute of Translational Pharmacology (IFT), National Research Council (CNR), 00133 Rome, Italy; antonio.mastino@ift.cnr.it

**Keywords:** West Nile virus, antiviral peptides, frog-derived peptides, AR-23, RV-23, viral entry inhibition, molecular docking

## Abstract

Over the past five decades, the emergence and re-emergence of multiple flaviviruses have triggered significant global outbreaks, posing serious threats to public health. Among them, West Nile virus (WNV) is a major cause of mosquito-borne illness, typically presenting as an acute systemic febrile disease and, in some cases, progressing to the central nervous system involvement. No specific antiviral therapies or effective vaccines are available for WNV infections. In this context, antimicrobial peptides (AMPs) with antiviral properties—known as antiviral peptides (AVPs)—have gained attention as potential therapeutic agents due to their ability to interfere with various stages of the viral life cycle. Two frog-derived melittin-like peptides, AR-23 and RV-23, were synthesized and purified, and their hemolytic activity was assessed on human erythrocytes. Antiviral activity against WNV was evaluated in Vero cells using cytopathic effect reduction assays and real-time PCR quantification of viral RNA. Time-of-addition experiments were conducted to explore the stage of viral inhibition. In silico molecular docking studies were performed to examine interactions between the peptides and the viral E glycoprotein. Both peptides displayed strong antiviral effects during the early phases of infection, likely through direct interaction with viral particles and disruption of virus–host interactions. Compared with melittin, AR-23 and RV-23 showed greater efficacy and lower cytotoxicity, highlighting their potential as promising therapeutic candidates for flavivirus infections.

## 1. Introduction

Emerging viral infections remain a major public health challenge due to their potential to cause widespread epidemics and pandemics. In recent decades, diseases caused by viruses of the *Flavivirus* genus have shown a marked increase in global incidence, raising substantial medical, veterinary, and epidemiological concerns [1]. Flaviviruses constitute a large group of positive-sense, single-stranded RNA viruses primarily transmitted by arthropod vectors, such as mosquitoes and ticks. This genus comprises several emerging or re-emerging pathogens of critical importance, including Zika virus (ZIKV), yellow fever virus (YFV), Japanese encephalitis virus (JEV), Usutu virus (USUV), and West Nile virus (WNV) [2,3,4]. A range of factors, including uncontrolled urbanization, climate change, the expansion of vector habitat, and increased international travel, have amplified the epidemic potential of these viruses [5,6]. WNV has gained particular attention due to its widespread geographical distribution and its involvement in recurrent outbreaks, especially across Europe, the Americas, and parts of Africa and Asia [7,8]. In Europe, recurrent outbreaks have been reported in Southern and Central-Eastern regions, positioning WNV as a persistent threat to human and animal health [8]. As of the latest epidemiological report from the National Institute of Health (2025), approximately 32 confirmed cases of WNV infection have been reported in Italy. Notably, most of these cases have manifested in the more severe neuroinvasive form of the disease, underscoring the public health implications of the current outbreak [9,10]. Alarmingly, two fatalities have also been documented, both occurring in the Central-Northern regions of Lazio and Piemonte, highlighting the potential lethality of WNV and the need for continued surveillance and preventive measures [11,12]. WNV is a flavivirus maintained in nature through an enzootic transmission cycle involving birds as primary reservoirs and *Culex* mosquitoes as vectors. Humans and horses are incidental, dead-end hosts; however, infection can lead to severe clinical outcomes such as encephalitis, meningitis, and acute flaccid paralysis, particularly in immunocompromised or elderly individuals. Despite its considerable public health impact, there are currently no specific antiviral treatments or approved vaccines for WNV, suggesting the urgent need for continued epidemiological surveillance and the development of effective preventive strategies [13].

In this scenario, antimicrobial peptides (AMPs) have garnered increasing attention for their broad-spectrum activity against various microorganisms, including bacteria, fungi, parasites, and viruses [14]. As evolutionarily conserved components of the innate immune system, AMPs are present across diverse biological kingdoms, including bacteria, plants, insects, reptiles, and, most notably, amphibians [14]. Amphibians, which often inhabit hostile and pathogen-rich environments, secrete AMPs from dermal glands through a holocrine mechanism as a part of their innate defense strategy [15]. These peptides, typically consisting of 10–50 amino acids, are relatively easy to synthesize, exhibit low toxicity towards host cells, and demonstrate high efficacy and selectivity [16]. In contrast to conventional small-molecule drugs, AMPs target multiple components within pathogens, thereby minimizing the risk of resistance development.

In addition to their well-documented antibacterial properties, many amphibian-derived AMPs exhibit potent antiviral activity. These antiviral peptides (AVPs) typically exert virucidal effects by directly interacting with and neutralizing virus particles. For instance, temporin L, a peptide isolated from the skin of the European red frog (*Rana temporaria*), and its synthetic analogs have demonstrated significant inhibitory activity against a broad range of enveloped viruses, including herpesviruses, paramyxoviruses, influenza virus, and coronaviruses such as severe acute respiratory syndrome coronavirus 2 (SARS-CoV-2) [16]. The antiviral activity of temporins is primarily attributed to their ability to interact with and disrupt viral envelopes, thereby preventing virus entry into host cells. This envelope-targeting mechanism is supported by the lack of activity observed against non-enveloped viruses, indicating a degree of specificity. Beyond direct virucidal action, a few amphibian AVPs have also been reported to inhibit infection by targeting intracellular processes, such as interfering with viral replication machinery or blocking genome replication. For example, dermaseptins are a family of peptides isolated from *Phyllomedusa* frogs, known for blocking human immunodeficiency virus 1 (HIV-1) replication by specifically targeting reverse transcriptase activity [17]. Similarly, caerins from *Litoria caerulea* can penetrate host cells and block intracellular stages of HIV-1 infection by interfering with viral integrase and other replication enzymes [18,19].

In this study, we investigated the antiviral activity of AR-23 (derived from *Rana tagoi*) and RV-23 (from *Rana draytonii*) against WNV infection. Both peptides possess well-characterized antimicrobial properties [15,20,21,22,23]. Specifically, AR-23 has demonstrated broad-spectrum antiviral activity, effectively inhibiting enveloped viruses, including herpesviruses, paramyxoviruses, bunyaviruses, and coronaviruses. Its antiviral mechanism primarily targets the early stages of viral entry, as evidenced by significant reductions in viral replication when the peptide is pre-incubated with the virus. In contrast, RV-23, which shares structural similarity with AR-23, has been less extensively evaluated for antiviral properties. Both peptides share strong structural and functional similarities with melittin (from *Apis mellifera*), a well-known antiviral peptide, used as a positive control in our experiments for a comparative study [24,25,26]. Our findings indicate that both peptides exert significant antiviral effects by directly targeting the early stages of the WNV replication cycle. These results highlight AR-23 and RV-23 as promising candidates for the development of peptide-based antiviral therapies.

## 2. Results

### 2.1. Evaluation of Peptides’ Cytotoxicity

**Figure 1:**
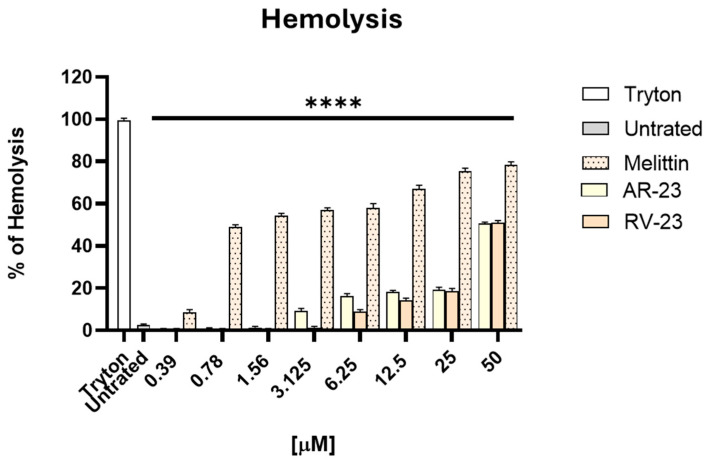
Evaluation of the hemolytic activity of AVPs. The toxic effect of peptides was investigated on human red blood cells. The percentage of hemolysis was examined, and MHC was identified. Tryton was used as a positive control, while untreated cells dissolved in PBS were the negative control. **** *p* < 0.0001.

The hemolytic activity of AR-23 and RV-23 was assessed using human erythrocytes and compared to that of melittin (Figure 1). 

AR-23 and RV-23 exhibited a 50% hemolysis concentration (HD_50_) of approximately 50 μM. In contrast, melittin showed markedly higher hemolytic activity, with an HD_50_ value below 1 μM. Importantly, AR-23 and RV-23 did not induce significant hemolysis at concentrations below their respective MHCs, indicating a more favorable safety profile than melittin. The cytotoxicity of these peptides on Vero cells has been reported previously [23], and the concentrations used in the subsequent assays were below both the cytotoxic levels and the HD_50_.

### 2.2. Antiviral Properties of Peptides

**Figure 2:**
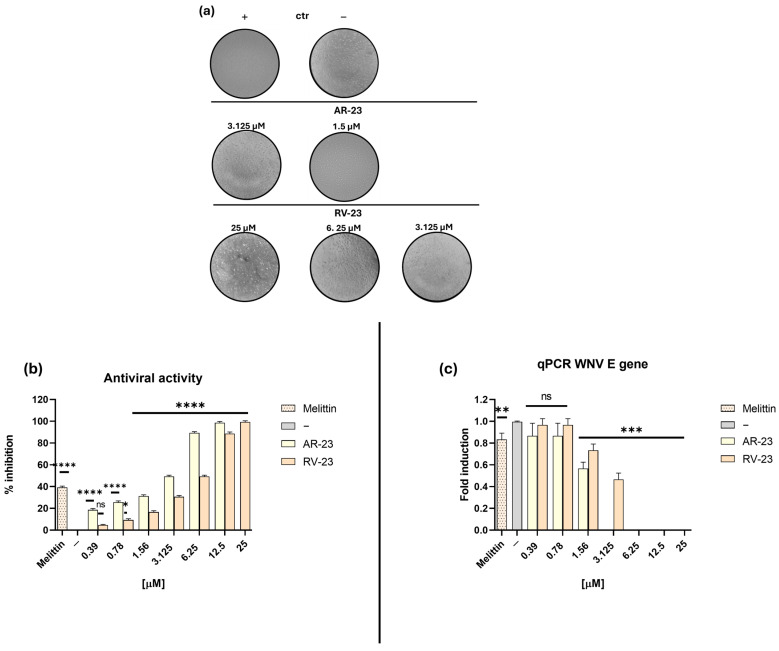
Evaluation of the inhibitory activity of AR-23 and RV-23 against WNV in the virus pre-treatment assay. (**a**) Representative images of the CPE following exposure to increasing concentrations of AR-23 (0.39–12.5 µM) and RV-23 (0.39–25 µM). (**b**) Quantification of WNV inhibition (%). After 1 h of incubation of the virus–peptide mixture at 37 °C, 100 μL of complete medium was added. (**c**) Molecular assay: Real-time PCR was performed to investigate the effect of AR-23 and RV-23 on the expression of the *E* viral gene. Melittin (1.56 μM) was included as a positive control (+), while the negative control (−) is represented by infected but untreated cells. Data represent the mean ± SD of three independent experiments. **** *p* < 0.0001; *** *p* = 0.0008; ** *p* = 0.0033; * *p* = 0.0295; ns: non-significant.

Non-cytotoxic concentrations of AR-23 and RV-23 were used to evaluate their potential inhibitory activity against WNV infection, and their effects were compared to melittin. A plaque reduction assay was performed in which each peptide (0.39-12.5 µM for AR-23, and 0.39-25 µM for RV-12) was incubated with the virus (MOI 0.1), and the resulting mixture was then applied to Vero cell monolayers. As shown in Figure 2a,b, both AR-23 and RV-23 demonstrated significant antiviral activity against WNV, suggesting interference with the early stage of the viral life cycle.

AR-23 and RV-23 inhibited viral replication with 50% inhibitory concentration (IC_50_) values of 3.125 and 6.25 µM, respectively, while melittin exhibited a higher IC_50_ of 10 µM. In contrast, neither AR-23 nor RV-23 showed activity in cell pre-treatment or post-treatment assays, indicating that their antiviral effect is not mediated through interaction with the host cell membrane or intracellular replication processes (Figure S1).

To further confirm the antiviral activity of AR-23 and RV-23, a molecular analysis was conducted via real-time quantitative PCR (RT-qPCR) to assess viral gene expression. In accordance with the virus pre-treatment protocol described in Section 4.4, WNV was incubated with each peptide before the infection, and total RNA was extracted from Vero cells 30 h post-infection. The expression of a key viral gene—the *E* gene (envelope glycoprotein)—was quantified. As shown in Figure 2c, both AR-23 and RV-23 significantly reduced *E* transcription levels in a dose-dependent manner. Complete suppression of gene expression was observed at peptide concentrations between 6.25 and 25 µM, indicating effective interference with early stages of the WNV replication cycle. Melittin, used as a positive control, only slightly inhibited viral gene expression, but its high hemolytic activity limits its therapeutic applicability compared to AR-23 and RV-23.

### 2.3. Molecular Docking Analysis

**Figure 3:**
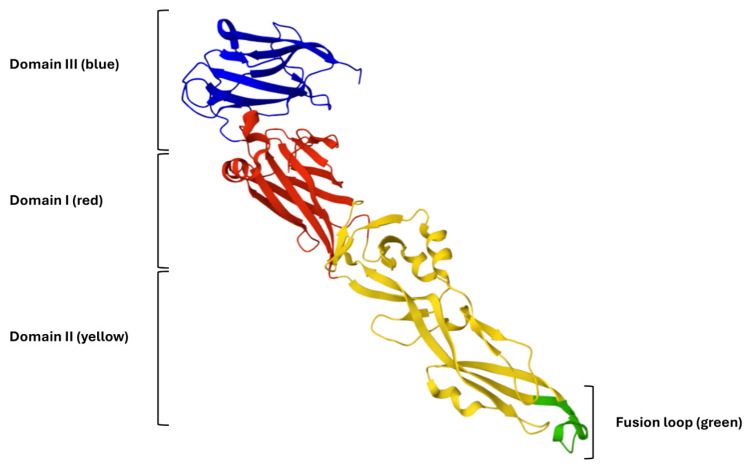
Three-Dimensional representation of the WNV E glycoprotein. Domain I (DI) is shown in red, domain II (DII) in yellow, and domain III (DIII) in blue. The conserved fusion loop at the distal tip of domain II is highlighted in green.

The E glycoprotein of WNV, a class II viral fusion protein, is essential for host cell attachment and membrane fusion. Its crystal structure (PDB ID: 2HG0) reveals a homodimeric arrangement at neutral pH, consisting of three distinct domains: domain I (DI), a central β-barrel; domain II (DII), an elongated structure containing the conserved hydrophobic fusion loop; and domain III (DIII), an immunoglobulin-like domain implicated in receptor binding (Figure 3) [27].

The hinge regions connecting the three domains of the E glycoprotein confer the flexibility required for the conformational rearrangements that drive membrane fusion. Notably, the fusion loop within DII is highly conserved and has been identified as a key target for viral entry inhibitors [28]. Based on this structural framework, synthetic inhibitory peptides may disrupt domain reorganization or directly bind the fusion loop, thereby preventing the fusogenic transition of the E protein. Consequently, in silico docking and structure-guided design approaches centered on the E glycoprotein represent promising strategies for the development of novel antiviral agents against WNV. In this context, we explored whether AR-23 and RV-23 can interact with glycoprotein E by performing molecular docking simulation analyses.

**Figure 4:**
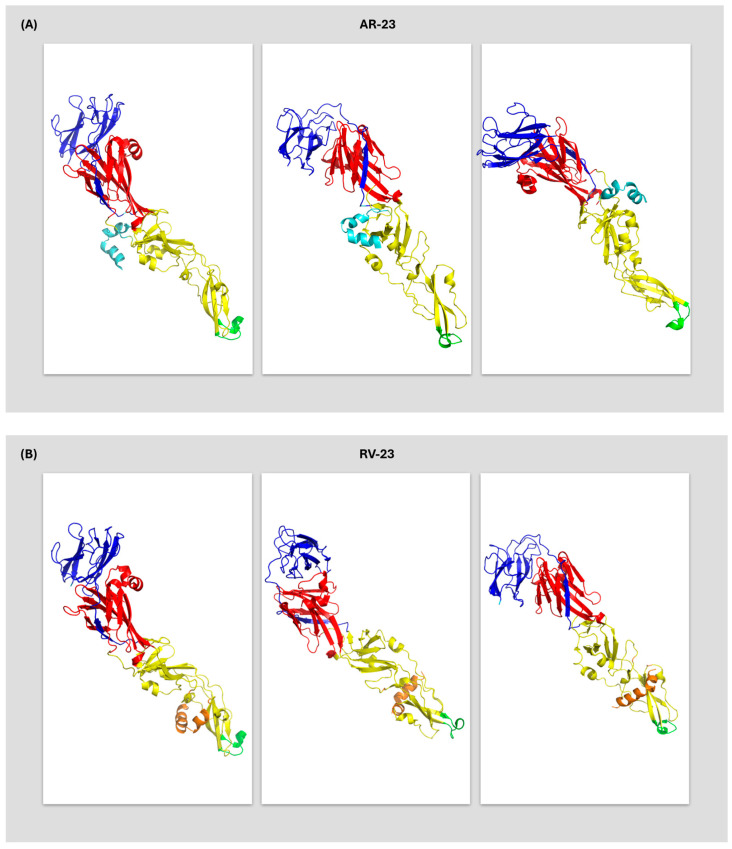
Three-Dimensional representation of the binding interaction between WNV E glycoprotein and peptides (**A**) AR-23 (indicated in cyan) interacting with DI and DII, and (**B**) RV-23 (indicated in orange) binding to DII. WNV E glycoprotein domains are represented as in Figure 4.

Molecular docking simulations were carried out using the ClusPro 2.0 online software (https://cluspro.org/peptide/index.php, accessed on 15 October 2025), which is well-suited for peptide-protein docking. The most energetically favorable conformation identified by the software, representing the highest predicted binding stability, is shown in Figure 4.

**Figure 5:**
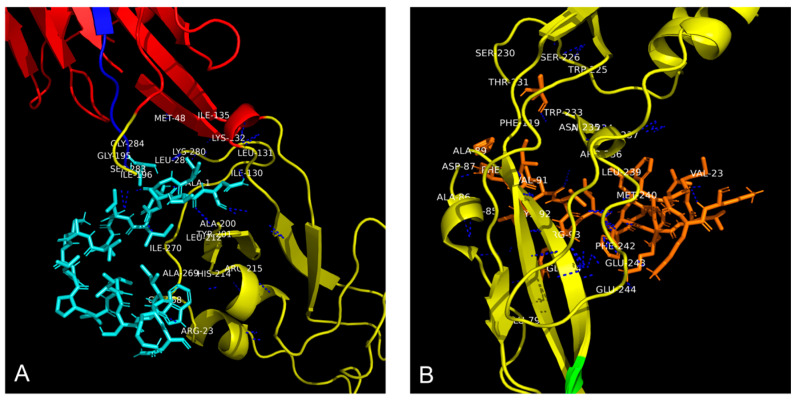
Residues involved in the interaction between WNV E glycoprotein and peptides (**A**) AR-23, indicated in cyan, and (**B**) RV-23, indicated in orange. Polar interactions were highlighted with blue dashed lines. WNV E glycoprotein domain I is represented in red, while domain II is reported in yellow. The amino acid residues not visible in the figure due to the three-dimensional rotation of the glycoprotein are: SER 283, LEU 281, and GLY 268 for AR-23; TRY 225, THR 231, ASN 235, ARG 236, GLU 237, GLN 94, GLU 79, ARG 93, CYS 92, ARG 85, and PHE 90 for RV-23.

Structural analysis of the complex formed between the synthetic peptide AR-23 and WNV E-glycoprotein (Figure 4A) revealed a multidomain binding pattern. The peptide establishes direct contacts within 4 Å with critical residues in both DI and DII (Figure 5A).

This interaction profile suggests that AR-23 may disrupt the E protein’s structural integrity and fusogenic function. The residues comprise polar amino acids, indicating the presence of hydrophilic interactions. Similarly, docking analysis of RV-23 in complex with WNV E glycoprotein demonstrated interactions with several residues primarily located in DII (Figure 4B). Residues within 4 Å of RV-23 were identified (Figure 5B), reinforcing the hypothesis that this peptide targets the fusion-associated region of the protein, potentially interfering with virus–host membrane fusion.

To estimate the binding affinity between the peptides and the WNV E glycoprotein, the PRODIGY (PROtein binDIng enerGY prediction) web server was employed. This bioinformatics tool calculates the binding free energy (ΔG) of protein–protein or protein–peptide complexes based on the structural characteristics of the interaction interface. Using the 3D models of the peptide–glycoprotein complexes, ΔG values were obtained for both AR-23 and RV-23. From these values, the inhibition constant (Ki) was calculated using the following thermodynamic equation:

ΔG = RT⋅ln(Ki)

where R is the universal gas constant (1.987 cal·mol^−1^·K^−1^) and T is the absolute temperature in Kelvin (typically 298 K).

Table 1 presents a comparative analysis of AR-23 and RV-23 based on their molecular docking parameters, including ΔG, Ki, and interaction interface characteristics.

Although both peptides exhibit strong binding affinities (AR-23: −14.3 kcal/mol; RV-23: −13.9 kcal/mol), the multidomain interaction profile of AR-23 provides a strategic advantage for viral fusion inhibition. By simultaneously engaging DI and DII of the E glycoprotein, AR-23 is likely to disrupt both the insertion of the fusion loop (located in domain II) and the conformational transitions mediated by DI. This dual interference may lock the glycoprotein in a prefusion, inactive state, effectively preventing viral entry into the host cell. In contrast, RV-23, while maintaining comparable binding, interacts more selectively with DII, suggesting a more localized mechanism of action. Such selectivity may confer advantages regarding target specificity, although it may not provide the same potency of functional disruption as AR-23.

## 3. Discussion

Flaviviruses pose an escalating global health threat due to the increasing number of reported cases and the lack of approved antiviral treatments or vaccines for many of these viruses, including WNV, which remains a priority pathogen of concern. This study investigated the antiviral potential of two melittin-like peptides, AR-23 and RV-23, derived from frog skin, against WNV infection. Melittin, a well-characterized AVP isolated from *Apis mellifera*, exerts its primary antiviral activity by disrupting lipid membranes. By inserting into and destabilizing viral envelopes, melittin induces direct lysis of several enveloped viruses [24,25,26,29,30]. However, the main limitation of melittin lies in its cytotoxicity to mammalian host cells, which significantly restricts its clinical applicability [25,31,32]. We have previously demonstrated that AR-23 and RV-23 possess a broad-spectrum antiviral activity against various human enveloped viruses, including herpesviruses, coronaviruses, paramyxoviruses, and influenza virus [33]. Their efficacy has also been confirmed against several animal viruses, such as bovine and caprine herpesviruses, canine distemper virus, and Schmallenberg virus [22,23]. In these studies, both peptides significantly reduced viral infectivity, primarily by interacting with viral surfaces, thereby preventing attachment and penetration into host cells.

Building on these findings, we extended the antiviral evaluation of AR-23 and RV-23 to flaviviruses. First, their hemolytic activity was analyzed, resulting in a similar value with an HD_50_ of 50 μM (Figure 1). It should be emphasized that both peptides show significantly lower cytotoxicity than melittin, highlighting the latter’s limited therapeutic potential due to its high toxicity. About the antiviral activity (Figure 2a,b), AR-23 displayed greater antiviral potency among the two, as indicated by its higher selectivity index (SI = 16) compared to RV-23 (SI = 8), and both peptides are more active than melittin (SI = 0.078). The mechanism of action of both AR-23 and RV-23 is probably targeted to the early stages of the viral replication cycle without compromising host cell membrane integrity or affecting intracellular replication pathways. The absence of inhibition in the pre-treatment condition is consistent with cells being washed before infection, leaving no peptide present during viral entry. Similarly, post-treatment with peptides had no measurable effect, likely because the analysis was performed before significant secondary rounds of infection occurred (30 hpi). These findings support the conclusion that AR-23 and RV-23 act primarily on extracellular viral particles at the stage of attachment/entry, but not on intracellular replication or spread. In addition, we also demonstrated that AR-23 and RV-23 provided a marked reduction in the expression of the *E* gene, which encodes a key envelope glycoprotein involved in viral entry (Figure 2c), reflecting a general inhibition of viral replication.

Antiviral peptides can exert virucidal effects by degrading the viral envelope, inhibiting virus–receptor interactions through saturation of the cell surface, or targeting viral proteins essential for entry into host cells. Targeting the latter mechanism represents a selective and promising strategy for developing novel antiviral agents for therapeutic use. In this framework, to explore the potential antiviral mechanism of AR-23 and RV-23, we performed a molecular docking analysis between peptides and the WNV E glycoprotein.

In silico docking and structure-guided design targeting the WNV E glycoprotein represent promising strategies for developing novel antiviral agents. The WNV E, a class II viral fusion protein, is critical for host cell attachment and membrane fusion. Its crystallographic structure (PDB ID: 2HG0; Figure 3) reveals a homodimeric organization at neutral pH and consists of three domains [27]. Flexibility between these domains, conferred by hinge regions, facilitates the conformational changes required for membrane fusion. Notably, the conserved fusion loop within DII has been identified as a key target for viral entry inhibitors [28,34].

Molecular docking analysis (Figure 4) revealed distinct binding profiles for AR-23 and RV-23 with the WNV E glycoprotein. AR-23 (Figure 4A) exhibited broader interactions, engaging residues across DI and DII. Such multidomain binding is strategically advantageous for inhibiting viral fusion, as it can simultaneously block multiple key functions of the E glycoprotein, including fusion loop insertion (DII) and interdomain conformational rearrangement (DI). This broad interaction could stabilize the E protein in an inert prefusion state, preventing the reorganization necessary for viral entry into the host cell. Energetic analysis (ΔG and Ki, Table 1) confirmed that both peptides exhibit high binding affinities for the WNV E protein. However, AR-23 showed superior binding energy and a lower inhibition constant, consistent with its stronger in vitro antiviral potency (IC_50_ = 3.125 μM) compared to RV-23 (IC_50_ = 6.25 μM). These in Silico and in vitro results suggest that AR-23 may exert its antiviral effect through a more potent and structurally disruptive interaction with the WNV E glycoprotein. Both AR-23 and RV-23 are amphipathic α-helical peptides structurally related to melittin. Compared with melittin (26 residues, net charge +6), AR-23 (23 residues, net charge +5) and RV-23 (23 residues, net charge +6) differ in amino acid composition and in the distribution of hydrophobic residues [20,23], which may contribute to their reduced hemolytic activity relative to melittin.

Melittin is known to exert broad-spectrum antiviral activity through disruption of lipid bilayers, leading to virucidal effects on enveloped viruses [35,36,37,38,39]. The structural similarity of AR-23 and RV-23 to melittin suggests that membrane interactions may also play a role in their antiviral activity, although their lower hemolytic potential indicates altered selectivity. In conclusion, we experimentally demonstrate that AR-23 and RV-23 exhibit greater antiviral efficacy and significantly lower cytotoxicity than melittin, increasing their potential as therapeutic candidates for flavivirus infections. Specifically, the in Silico analyses conducted in this study suggest that these peptides may interact distinctly with the WNV E glycoprotein, opening new avenues for their therapeutic use compared to melittin. The experimental findings of this study, however, do not provide a direct demonstration of the action of peptides on the virus. In fact, other mechanisms, including a direct virucidal activity of the peptides, could be involved in the anti-WNV activity of AR-23 and RV-23, and future research will need to address this aspect.

## 4. Materials and Methods

### 4.1. Preparation and Characterization of AVPs

The C-terminal amidated peptides RV-23 (H-RIGVLLARLPKLFSLFKLMGKKV-NH_2_) and AR-23 (H-AIGSILGALAKGLPTLISWIKNR-NH_2_) were synthesized using an optimized Fmoc solid-phase peptide synthesis protocol [33]. Melittin was acquired from Sigma-Aldrich (Saint-Louis, MI, USA). Cleavage of AR-23 from the solid support was performed by treating the resin with a mixture of TFA/TIS/H_2_O (95:2.5:2.5, *v*/*v*/*v*) for 3 h at room temperature. For the RV-23 peptide susceptible to Methionine oxidation, the cleavage was performed by adding 5% *w*/*v* amino acid Met to the TFA/TIS/H_2_O cleavage mixture as reported above for 3 h at room temperature. Crude peptides were subsequently precipitated using cold diethyl ether, dissolved in a solution of H_2_O/CH_3_CN (75:25 *v*/*v*), and then lyophilized.

Preparative purification of peptides was performed by high-performance liquid chromatography (HPLC) on a WATERS 2545 system (Waters, Milan, Italy), equipped with a WATERS 2489 UV/Vis detector. The gradient employed involved a linear increase from 5% to 70% in 20 min in the percentage of acetonitrile (CH_3_CN) containing 0.05% TFA in water with 0.05% TFA, at a 12 mL/min flow rate. Peptides were identified and characterized by Agilent 1290 Infinity LC ESI-TOF-MS mass spectrometry (MS) coupled to an Agilent 6230 time-of-flight (TOF) LC/MS system (Agilent Technologies, Cernusco sul Naviglio, Italy). Peptide LC-MS characterization was performed using a Waters xBridge C18 column (3 μm, 4.6 × 5.0 mm), applying a linear gradient of CH_3_CN/0.05% TFA in H_2_O/0.05% TFA, from 5 to 70% in 20 min, at a flow rate of 0.2 mL/min. The yields of purified peptides were estimated to be about 80% and the purity was up to 98%.

### 4.2. Hemolytic Assay

The hemolytic potential of these peptides was evaluated using freshly collected human erythrocytes. Briefly, 10 mL of whole blood was centrifuged at 500 rpm for 5 min (min) to separate and discard the plasma. The erythrocytes were washed thrice with 150 mM NaCl and then diluted 1:50 in phosphate-buffered saline (PBS). A 180 µL of the erythrocyte suspension was added to each 96-well U-bottom plate, followed by 20 µL of peptide solutions at concentrations ranging from 0.39 to 50 µM. Samples were incubated for 1 h (h) at 37 °C. PBS and 0.1% (*v*/*v*) Triton X-100 were negative and positive controls, respectively. Hemoglobin release, indicative of hemolysis, was measured by absorbance (*Abs*) at 540 nm using a spectrophotometer. The percentage of hemolysis was calculated using the following formula [40]:

$$\%\;hemolysis=\frac{\left[(Abs\;Peptide-Abs\;PBS)\right]}{\left[Abs\;Triton-Abs\;PBS\right]}\times 100$$

*Abs Peptide* is the absorbance of the peptide-treated sample, *Abs PBS* is the absorbance of the negative control, and *Abs Triton* is the absorbance of the positive control.

### 4.3. Cells and Viruses

A monkey kidney epithelial cell line (Vero, ATCC CCL-81, Manassas, VA, USA) was maintained in Dulbecco’s Modified Eagle Medium (DMEM, Microtech, Naples, Italy) containing 4.5 g/L D-glucose, and supplemented with 100 IU/mL penicillin/streptomycin (Himedia, Naples, Italy) and 10% fetal bovine serum (FBS, Microtech).

WNV was kindly provided by the Italian National Institute of Health (ISS, Rome, Italy) and propagated in Vero cell monolayers following established protocols [16]. All WNV-related experiments were conducted under Biosafety Level 3 (BSL-3) containment at the Regional Reference Center for Arboviruses, located within the Microbiology and Virology Unit of the University Hospital ‘P. Giaccone in Palermo, Italy.

### 4.4. Antiviral Activity by 50% Tissue Culture Infectious Dose (TCID_50_)

To determine the stage of the viral replication cycle at which the peptides exert their antiviral effects, time-of-addition assays were conducted under three different conditions: (i) Virus pre-treatment: peptides were incubated with the viral inoculum (1 h at 37 °C), and then the mixture was added the cells for an additional hour; (ii) Cell pre-treatment: cells were pre-incubated with the peptides for 1 h at 37 °C, then cell monolayer was washed with PBS followed by infection with the virus for an additional hour; (iii) Post-treatment: Peptides were added to the cells immediately after the 1 h viral adsorption phase at 37 °C.

Following each treatment, 100 µL of complete medium (DMEM supplemented with 5% FBS) was added to each well. The cytopathic effect (CPE) was monitored microscopically after 30 hpi and confirmed by staining with 0.5% crystal violet (CV). All experiments were performed in triplicate. The degree of viral inhibition was quantified by comparing the CPE observed in peptide-treated samples to that in the negative control (−, infected but untreated cells), while melittin was used as a positive control (+) at a concentration of 1.56 μM.

### 4.5. Molecular Analysis: Real-Time PCR

Non-cytotoxic concentrations of peptides (ranging from 0.78 to 25 μM) were evaluated in a virus pre-treatment assay. After 30 h of incubation, total RNA was extracted from treated and control cells using TRIzol reagent (Thermo Fisher Scientific, Waltham, MA, USA) and quantified with a NanoDrop spectrophotometer (NanoDrop 2000, Thermo Fisher Scientific). Complementary DNA (cDNA) was synthesized from 1 μg of RNA using the 5× All-In-One RT Master Mix (Applied Biological Materials, Richmond, VA, USA), followed by amplification through Real-time PCR. The expression level of the WNV envelope (*E*) gene was analyzed, and threshold cycle (Ct) values were normalized to the housekeeping gene glyceraldehyde 3-phosphate dehydrogenase (*GAPDH*). Relative mRNA expression was calculated using the 2^−ΔΔCt^ method. All experimental and synthetic details, including primer sequences, are provided in Table 2. Oligonucleotides were synthesized from Eurofins (Ebersberg, Germany).

### 4.6. Molecular Docking Studies

The chemical structures of AR-23 and RV-23 peptides were predicted using the PEP-FOLD3 online software (https://mobyle.rpbs.univ-paris-diderot.fr/cgi-bin/portal.py#forms::PEP-FOLD3, accessed on 5 July 2025). The resulting models were exported as PDB files and further refined using Discovery Studio 2024 (BIOVIA, v24.1.0.23298), where water molecules and irrelevant chains were removed. The three-dimensional (3D) structure of WNV E glycoprotein was retrieved from the Protein Data Bank (PDB ID: 2HG0). Molecular docking simulations were performed using the ClusPro 2.0 server to evaluate peptide–protein interactions. The resulting complexes were analyzed and visualized with PyMOL version 2.6 (Schrödinger, LLC, New York, NY, USA, open-source/educational version). The binding affinity between the peptides and glycoprotein E was estimated using the PRODIGY web server (https://rascar.science.uu.nl/prodigy/ accessed on 6 July 2025), which calculates the binding free energy (ΔG) based on interface contacts and solvent-accessible surface area. Protein residues near the peptide—within ~4 Å, the typical distance for hydrogen bonding or hydrophobic interactions—were identified. These interacting residues were visualized and annotated using the PyMOL graphical interface, based on the spatial orientation of their side chains relative to the peptide.

### 4.7. Statistical Analysis

All experiments were performed in triplicate, and results are presented as mean ± standard deviation (SD). Statistical analyses were conducted using GraphPad Prism software (version 8.0.1). One-way ANOVA followed by Dunnett’s multiple comparisons test assessed significance, with *p*-values ≤ 0.05 considered statistically significant.

## 5. Conclusions

AR-23 and RV-23 exhibit strong inhibitory potential against WNV, interfering with viral entry without affecting host cells in the concentration range tested. In silico analyses suggest that AR-23 has a broader mechanism of action, potentially by interacting and interfering with the functional domains of viral glycoprotein E. RV-23, on the other hand, appears to act through a more selective and localized interaction pattern, which may offer advantages in terms of target specificity and fewer off-target effects on cellular proteins. Experimental validation will be necessary to clarify these mechanisms and fully evaluate the therapeutic potential of AR-23 and RV-23.

## Figures and Tables

**Table 1:** Binding characteristics of AR-23 and RV-23 peptides.

AVPs	Characteristics			
	Binding ΔG (kcal/mol)	K_i_ (hypothetical) (pM)	Domains involved	Residues
**AR-23**	–14.3	32	DI, DII	45
**RV-23**	–13.9	65	DII	43

**Table 2:** Sequences of the WNV genes analyzed in the present study.

GENE	SEQUENCE (5′→3′)
*E*	F: GCAACATGGGTGGATTTGGT
	R: GGGTCAGCACGTTTGTCATT
*GAPDH*	F: CCTTTCATTGAGCTCCAT
	R: CGTACATGGGAGCGTC

F: forward primer; R: reverse primer. *E*: envelope protein; *GAPDH*: glyceraldehyde 3-phosphate dehydrogenase.

## Data Availability

The data presented in this study are available on request from the corresponding authors.

## Supplementary Materials

The following supporting information can be downloaded at https://www.mdpi.com/article/10.3390/ijms262010148/s1.
