# Supplementary material for: Structure–Function Insights into Frog Skin Peptides Reveal Potent Inhibition of West Nile Virus Entry
Source: Int J Mol Sci. 2025 Oct 18;26(20):10148. doi: 10.3390/ijms262010148 (PMC12563094; doi:10.3390/ijms262010148)
Supplement: Supplementary file 1 [file ijms-26-10148-s001.zip › ijms-3882413-supplementary.pdf]

International Journal of *Molecular Sciences*

MDPI

Article

Structure–Function Insights into Frog Skin Peptides Reveal Potent Inhibition of West Nile Virus Entry

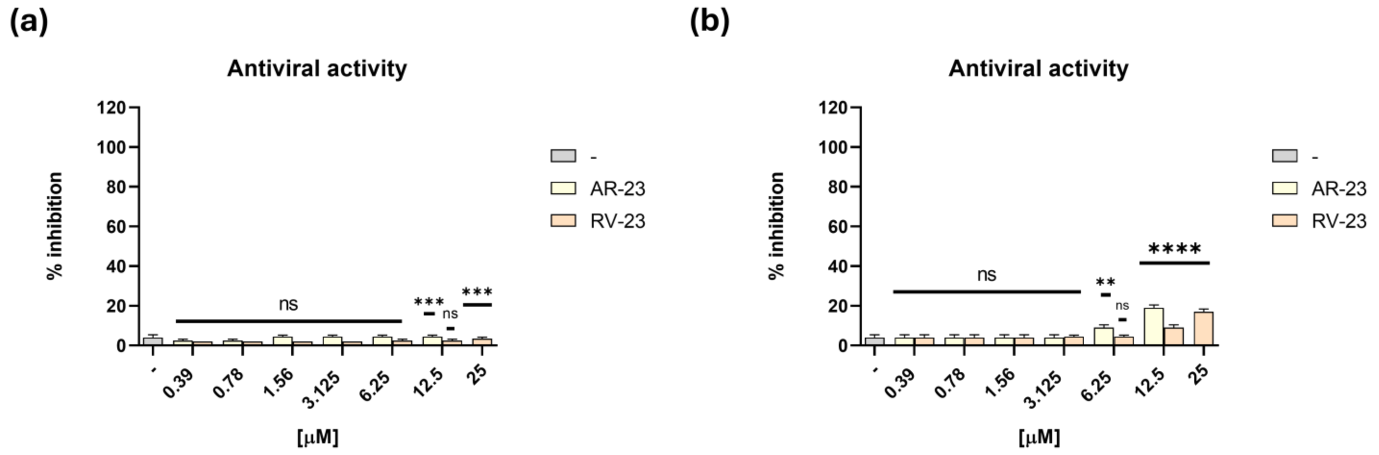

Figure S1. Evaluation of the inhibitory activity of AR-23 and RV-23 against WNV in cell pre-treatment and post-treatment assays. Increasing concentrations of AR-23 (0.39–12.5 µM) and RV-23 (0.39–25 µM) were tested in cell pre-treatment (a) and post-treatment (b) assays. Data represent the mean ± SD of three independent experiments. **** $p < 0.0001$; ***p=0.0008; **p=0.0055; ns: non-significant.
